# Predicting the Quality of Pasteurized Vegetables Using Kinetic Models: A Review

**DOI:** 10.1155/2013/271271

**Published:** 2013-09-26

**Authors:** Muhammad Aamir, Mahmoudreza Ovissipour, Shyam S. Sablani, Barbara Rasco

**Affiliations:** ^1^School of Food Science, Washington State University, Pullman, WA 99164-6376, USA; ^2^Department of Biological System Engineering, Washington State University, Pullman, WA 99164-6120, USA

## Abstract

A resurgence in interest examining thermal pasteurization technologies has been driven by demands for “cleaner” labeling and the need of organic and natural foods markets for suitable preventive measures to impede microbial growth and extend shelf life of minimally processed foods and ready-to-eat foods with a concomitant reduction in the use of chemical preservatives. This review describes the effects of thermal pasteurization on vegetable quality attributes including altering flavor and texture to improve consumer acceptability, stabilizing color, improving digestibility, palatability and retaining bioavailability of important nutrients, and bioactive compounds. Here, we provide kinetic parameters for inactivation of viral and bacterial pathogens and their surrogates and marker enzymes used to monitor process effectiveness in a variety of plant food items. Data on thermal processing protocols leading to higher retention and bioactivity are also presented. Thermal inactivation of foodborne viruses and pathogenic bacteria, specifically at lower pasteurization temperatures or via new technologies such as dielectric heating, can lead to greater retention of “fresh-like” properties.

## 1. Introduction to Thermal Processing Operations

Thermal processing involves heating of a food product at a temperature that ranges from 50 to 150°C primary to inactivate microbes and endogenous enzymes. The process chosen depends upon pH, microbial load, and desired shelf life [1]. Thermal processing includes pasteurization, commercial sterilization, operations for food tenderization, and thermal pretreatments such as blanching that are conducted prior to freezing and canning to inactivate bacteria and enzymes and remove entrapped air [2]. Thermal processing operations are conventionally classified according to the intensity of heat used: pasteurization (65–85°C), sterilization (110–121°C), and ultrahigh temperature (UHT) treatment (140–160°C). Enzyme reaction rates and microbial growth increase with temperature up to a certain limit at which point inactivation begins. While thermally processed products are treated is necessary if microbes of public health significance are to be controlled, the application of heat to fresh vegetables can cause severe quality deterioration, including degradation in color and texture, nutrient loss, cook loss, and area shrinkage. Furthermore, consumer demands thermally processed food in which important nutritive compounds are damaged as little as possible [3].

Pasteurization is a relatively mild heat treatment killing vegetative cells of pathogenic microorganisms that impact food safety. The level of heat treatments involved with pasteurization also inactivates enzymes limiting the impact of the deleterious reactions they cause on food quality. In the past, *Mycobacterium tuberculosis* was the widely accepted target microbe for pasteurization because its the importance in controlling disease causing microbe in milk, but recently *Listeria monocytogenes *has been replaced it in many applications as the target microbe for pasteurization of meats, seafood, and vegetables. Since the heat treatment in pasteurization is not severe enough to inactivate *Clostridium botulinum* spores, pasteurized foods require immediate refrigeration as a control with these requirements being particularly important for items such as vegetables that generally have a pH greater than 4.6 and water activity that is higher than 0.92. Thermotolerant spoilage microbes as well as bacterial spores survive pasteurization and can grow and cause deterioration of pasteurized products even when they are refrigerated, resulting in a shelf life for these foods of about one month or less depending on the composition. This is substantially less than the shelf life of commercially sterile foods which is one to three years. The optimal thermal process for pasteurization depends upon the nature of the food product, pH, microbial load, and resistance of target microorganisms; generally a number of combinations of time-temperature relationships can be equally effective for microbial control. Selection of the optimal process is usually dependent upon which will provide the best sensory properties and overall quality for the pasteurized food. 

**Figure 1:**
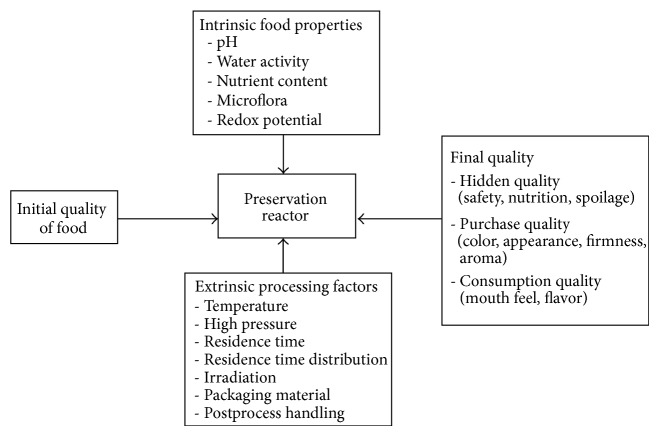
The preservation reactor: intrinsic and extrinsic factors that can influence the rate of deterioration of the quality of the product (adapted from [4]).

Determining what an adequate thermal process is has been transformed from an art to a science. Quantitative predictions of the impact of a particular thermal process in terms of safety and quality have become more common. Kinetic models can be developed to predict important parameters for process and equipment design, process optimization, monitoring, process verification, and control. These are then experimentally validated. Safety and quality are the two most important parameters of a food product and a summation of the effect of these combined reactions for a particular food product from production to the consumption. One model that summarizes these various characteristics is the “preservation reactor” presented in (Figure 1) [4]. 

This preservation reactor model applies to the entire product cycle, preparation, packaging, processing, distribution, and storage [4] and is appropriate for modeling thermal processes and the interrelationship of the extrinsic and intrinsic factors these processes have on the quality and safety of a pasteurized food. The time-temperature combination associated with a particular pasteurization process will control to a major extent the chemical, biochemical, and microbiological changes that will occur in a food product [4] and how alteration in the time-temperature relationship can influence both the desirable and undesirable reactions occurring during pasteurization, for example, undesirable browning that would occur at higher but not at lower pasteurization temperatures; also loss of heat labile nutrients in a longer compared to a shorter process. Among the extrinsic factors, temperature is usually considered to be the most vital factor to ensure safety and quality during the production and subsequent storage [5], but this notion should be replaced with an assessment of total heat associated with a thermal process. 

Thermal processes involve different unit operations so that the desired eating quality, safety, and shelf life for a particular food is obtained, for example, dehydration, blanching, pasteurization, sterilization, and cooking [6]; for this review, pasteurization is the primary focus, although data will also be provided for commercial sterilization processes for completeness an comparative purposes. Regardless of the process employed, understanding the intrinsic physical and thermal properties of the food is necessary, at least empirically, to design, optimize, and control a food production process over the range of 50–150°C, the most range important for food processing operations [7]. Thermophysical properties such as specific heat, enthalpy, thermal conductivity, thermal diffusivity, and heat penetration are dependent upon the chemical composition and structure of a food product and can vary with temperature. 

Pasteurization and sterilization are designed specifically to inactivate or destroy enzymes and microorganisms in foods. Cooking is a thermal process that is conducted by either the manufacturer or consumer to yield a food item with particular quality attributes while also contributing to improved safety. Dehydration may also help to improve the safety of the food product since a thermal treatment is usually involved, but microbial control in a dehydrated food is through the removal of water, reducing the amount available for microbial growth. Recent research on nonthermal processing methods including high pressure processing and pulse electric treatments alone or in combination with thermal processing show promise for juices and purees and whole tissue to a lesser extent. Combined thermal and nonthermal treatments can have a less detrimental impact on the sensory and nutritional properties of foods enhancing consumer acceptability [8, 9] either through greater sensory quality or an increase in perceived product safety than a nonthermal treatment alone. High pressure often causes textural changes and may not be effective in deactivating important spoilage microbes. Pulsed electric field treatments tend not to be effective for microbial control in solid or nonhomogeneous foods. Regardless, more research is needed in the area of combined treatments employing nonthermal methods particularly on microbial resuscitation following processing and inactivation of bacteria or pressure-stable enzymes in vegetable foods. Pasteurization can provide one of the hurdles for microbial control but is not enough by itself to maintain the safety and quality of pasteurized vegetables. Often, additional hurdles are used to control microbes such as reduction of water activity, change in pH, change in redox potential by incorporation of certain food additives, and use of preservatives [10]. 

## 2. Requirements for Vegetable Pasteurization Processes 

Maintaining fresh-like quality is an important feature in vegetable processing. Quality is a human construct comprising many food characteristics encompassing sensory properties (appearance, texture, taste, and aroma), nutritive values, presence or absence of specific chemical constituents, functional properties, and defects [8, 9]. The major concern consumers have with thermal processing of vegetables is retention of maximal sensory and nutritional quality. Changes in sensory and nutritional quality can occur at either a faster or slower rate than microbial inactivation [11, 12]. Unfortunately, even a mild thermal process tends to cause a significant loss of color and changes to texture, flavor, and potentially nutritive value. Because the basic objective of a thermal process is to provide a safe high quality food with respect to microbial load and presence of pathogenic microorganisms and inactivation of deleterious enzymes, these factors need to be balanced against the maintenance of nutritional quality including retention of bioactive components such as antioxidants. 

To optimize the time-temperature combination for thermal processing, many factors need to be considered, such as the type of the food, type of microorganism and microbial load, chemical composition of food material, nutrient value, and reaction kinetics for microbial death. The morphological and chemical composition of vegetables is different from fruits, requiring different thermal processing conditions. Clearly, vegetables vary greatly in their biological functions [13, 14]. 

Vegetables represent intact structural plant tissues, for example, celery, bamboo, and spinach, reproductive tissue such as peas and corn, and nutrient storage tissues represented by carrots, beets, and potatoes. Fruits for the most part are seed containing reproductive tissues of plants.

It is well understood that the main purpose of thermal processing is to inactivate pathogenic microorganisms and endogenous enzymes that make the food unfit for human consumption, but another important consideration is with the retention of nutrients, specifically vitamins as well as less studied nutritive component that may have antioxidative properties, anticarcinogenic or antiadiposity factors, and natural antimicrobial activity. Little work has been done in this area with the exception of studies on heat labile vitamins and some antioxidants. Certain heat sensitive nutrients such as ascorbic acid and thiamine are reduced [10] during thermal processing, and these two compounds are commonly used to monitor quality changes: vitamin C for pasteurization and some drying operations, and thiamine for higher heat processes including canning. Thermal effects on color and texture are commonly accepted and for some foods such as canned mushrooms and tomato juice are desirable despite the impact these might have on nutrients. 

Vegetables are generally low acid foods (pH > 6.0) with the notable exception of tomatoes (Table 1). The higher pH limits the diversity of available pasteurization processes for vegetables, juices, and purees [15–17], since a more severe heat treatment would be required to reduce the survival of and risk of subsequent growth of *Listeria monocytogenes* than for lower pH fruits and fruit juices. Vegetable purees have a higher buffering capacity than juice, and this also tends to increase the severity of the thermal process. However, to maintain quality, attempts are made to use as mild a thermal process as possible to maintain sensory quality and color. Despite the need for improved thermal processes as an option available for vegetable processing, there has been relatively little systematic research conducted on pasteurization of vegetable products generally, and the information that is available has emphasized higher temperature processes such as blanching and quick cook treatments. 

Furthermore, vegetables tend to contain both a greater variety and a higher concentration of heat resistant soil microorganisms than fruits. Therefore, vegetables products almost always require a more severe heat treatment to microbial spores. Strategies to reduce the level of soil microbes on vegetables through cultivation techniques such as plastic mulching which limits the airborne dispersion of soil microbes onto the edible portion of the plant or staking which limits direct contact of the plant with the soil may reduce microbial contamination [18].

 Maintaining plant tissue in an intact form is important for its chemical and biochemical stability as a food product. The presence of membrane-bound organelles within plant cells compartmentalizes cellular functions [14]. When membranes are mechanically damaged, deleterious biochemical reactions can occur. As cell membranes begin to deteriorate after the plant is harvested, membranes becomes more permeable [19, 20] allowing for diffusion of cellular components and leading to quality loss resulting from biochemical reactions. Both of these phenomena will occur during thermal processing to some extent, and a knowledge of how cell structure could change during processing is important if we are to attempt to lessen the impact of processing on both the structural integrity of a plant based food as well as maintain sensory quality that is lost due to changes in flavor and color that result from membrane damage. Quantification of the degree of cellular disruption will allow for a comparison to be made between different processes and also for process optimization [21, 22]. 

## 3. Thermal Inactivation Kinetics Associated with Quality Changes

To model quality and safety changes occurring in foods during thermal processing, predictive models are often employed and can be of different reaction orders (zero-order, first-order, and second-order) or follow a Weibullian power law model [3]. Degradation kinetics of components tied to food quality attributes can be explained by such mathematical models [13] and have been shown to be effective tools for calculating the rate of chemical and biochemical reactions occurring either in homogenous liquid or semisolid foods during thermal processing and storage. These models provide useful data engineering design and process optimization. Models developed for microbial inactivation for different time-temperature combinations (TTCs) (discussed in greater detail in a later section) can be correlated with models that predict changes in food product quality [4, 13] following processing and during subsequent storage. However, the kinetic parameters predicted from mathematical models such as reaction order, rate constants, and activation energy should be experimentally validated before they are used to reliably predict microbial lethality for a commercial food process. 

Various models can predict thermally induced loss of nutrients, enzyme inactivation, and changes to color, flavor, and texture. This rate of conversion depends upon many factors, such as temperature, moisture, acidity, reactant concentration, packaging, and packaging properties [4]. The reaction rate equation for *n*th-order reaction is given by

(1) $$\begin{array}{c} \frac{dC}{dt}=-kC^{n}, \end{array}$$

where *C* is the concentration of reactant at any time *t*, *k* is the reaction rate constant, with unit (concentration)^1−*n*^/(time), and *n* is the order of the reaction. The negative sign represents a decrease in concentration with time [4].

In terms of concentration, (1) is generally expressed as follows:

(2) $$\begin{array}{c} C^{1-n}-{C_{0}}^{1-n}=\left(n-1\right)kt,\;n>1, \end{array}$$

where *C*_0_ is the concentration of reactant at zero time.

The reaction rate is actually represented by zero-, first-, and second-order reaction kinetics which are

(3) $$\begin{array}{c} \text{Zero order:}\;\;C-C_{0}=-kt, \end{array}$$

(4) $$\begin{array}{c} \text{First order:}\;\;\ln\left(\frac{C}{C_{0}}\right)=-kt, \end{array}$$

(5) $$\begin{array}{c} \text{Second order:}\;\;\frac{1}{C}-\frac{1}{C_{0}}=kt, \end{array}$$

where *C* is the concentration of a nutritive component, viable microorganisms, or component associated with a specific quality factor at time *t*, *C*_0_ is the initial concentration, and *k* is reaction rate constant (1/min). Sometimes fractional reaction orders have been observed for changes in quality parameters in foods. Care must be taken when conducting experiments to determine or validate the reaction order since the resultant models need to be correct if these are to be used to develop accurate thermal processes [4, 23].

An important factor in the development of models for thermal processing of foods is to understand the temperature dependence of the reaction in question. The rate constant (*k*) is temperature dependent and is described by the Arrhenius equation shown here:

(6) $$\begin{array}{c} \ln k=\ln A-\frac{E_{a}}{RT}, \end{array}$$

where *A* is a preexponential factor (1/sec), *E*_*a*_ is activation energy (kJ/mol), *T* is the temperature (K) and *R* is the universal gas constant (8.314 J/(mol.k)). 

Activation energy is the minimum amount of energy required to initiate a reaction and is commonly calculated from a regression equation of ln(*k*), versus the reciprocal of absolute temperature (1/*T*). The magnitude of *A* varies from 10^14^ to 10^20^ sec^−1^ for unimolecular reactions and from 10^4^ to 10^11^ sec^−1^ for bimolecular reactions. As examples of the importance of these parameters in models for food quality, kinetic parameters associated with quality attributes for a number of vegetables are presented in Table 2. 

## 4. Factors Affecting Heat Transfer during Vegetable Pasteurization

Thermal processing involves the transfer of heat from the surface to the interior of the food. Heat transfer in foods is typically by conduction, convection, or radiant heat. Solid foods are heated from the external surface to the interior by conduction. Because mixing is possible for liquids, both conduction and convection may be involved [24]. If a phase change occurs during thermal process, for example, conversion of water to steam, the heat associated with the phase change should also be factored in when calculating a thermal process. Penetration of heat into the center of the food product and determination of the “cold spot” for food products heated in containers is controlled in part by the resistance to heat transfer within the product which is a function of the thermal conductive properties intrinsic to the food and the size and geometry of the food material. 

For foods heated in containers, the heat transfer at the boundary of the container and the heating medium and then between the container and the food [25] must also be taken into consideration, although in most cases the contribution of these two factors to the overall heating of the food is relatively small. Heat transfer through the container wall is by conduction. For metallic container of normal thickness and thermal conductivity, there is no appreciable resistant to heat transfer. Heat transfer from the container wall into the food depends upon the viscosity and thermal conductivity of the liquid component that is in contact with both the container wall and the solid food. The liquid at this interface is heated by both conduction and to a lesser extent by convective heating. 

For heterogeneous foods, heating is by a combination of conduction and convection and making their heating behavior difficult to model. For vegetables, the brine or liquid fraction is heated primarily by convection and the particulate matter by conduction [26–28]. One example of how conduction and convective heating could play a role in heating processes of the one vegetable in different product forms is described for mushrooms showing how packing density, shape, and particle orientation can affect heating. A conductive heating model would apply to slices that are densely packed. A combination of conduction and convection would apply to a container of smaller fragments or dices that are not tightly packed. Whole mushrooms would heat primarily by conduction. Models for heterogeneous foods such as this require that the thermal conductivity of both the brine and the vegetable be known. A temperature distribution within the brine and within large food particles should also be determined so that an appropriate model for the thermal process can be developed. Regardless of the type of food to be processed, knowing the heat transfer coefficients is important for kinetic models and when models are being developed to predict product temperature distribution during processing [29–31]. 

A number of models for heat transfer in foods have been developed that are applicable to conductive heating of vegetable foods. Many of these models emphasize changes in an important quality parameter such as texture as subjective criteria for assessing the thermal process. Experiments to validate the effectiveness of conductive heating processes are recommended but how these experiments are conducted will determine the applicability of the results obtained for the related food items. Selection of an appropriate kinetic model for textural changes can depend upon whether the heating is conducted under steady-state (isothermal heating) or non-steady-state (nonisothermal heating) conditions [32]. For short thermal processes this distinction is important. As an example, for longer heating times, the force required to fracture asparagus was similar under steady-state and non-steady-state heating methods. However, a non-steady-state method at a shorter heating time for asparagus resulted texture degradation when compared to steady-state method. Similar results have been found for other intact vegetables with a cylindrical geometry, for example, whole cucumbers, whole carrots, and corn-on-the-cob [33]. High-temperature short-time (HTST) processes are widely employed [25, 26]; non-steady-state processes and the steep thermal gradients and rapid heat transfer rate provide an advantage for heating many solid and viscous foods [34] but not necessarily intact plant tissue.

## 5. Microbial Thermal Inactivation 

Thermal death rate kinetics of microorganisms must be studied to optimize time-temperature combinations of a thermal process to obtain the desired lethality. Most research support the proposition that inactivation or degradation of microorganisms follows first-order reaction kinetics (4), represented as follows:

(7) $$\begin{array}{c} \ln\left(\frac{N}{N_{0}}\right)=-k\cdot t, \end{array}$$

where *N*_0_ represent number of viable microorganisms at time zero and *N* at time *t* and *k* is the reaction rate constant.

By the use of equation (6) for the thermal inactivation kinetics of microorganisms at reference temperature *T*_ref_  and reference reaction rate constant *k*_ref_, as in the following:

(8) $$\begin{array}{c} \ln k=\ln k_{\text{ref}}-\left[\left(\frac{E_{a}}{R}\right)\left(\frac{1}{T}-\frac{1}{T_{\text{ref}}}\right)\right]. \end{array}$$

The activation energy (*E*_*a*_) for bacterial spores has been reported in a range of 217–513 kJ/mol [35, 36]. This high magnitude of activation energy has been explained in various ways by many researchers, and a modified form of the Arrhenius model has been proposed to describe nonlinear forms of microbial inactivation curves. 

## 6. Microbial Inactivation

### 6.1. Temperature Sensitivity of Microbial Inactivation

The temperature sensitivity of *D*-values is measured as a *Z*-value which represents the influence of temperature on *D*-values. *Z*-value is a thermal resistant constant and can be defined as the increase in temperature causing 90% reduction in *D*-value. All microorganisms have different *Z-*values, and this value can be affected by a number of environmental factors for the same species such as pH, water activity, product form and dimensions, type and level of nutrients, buffering capacity, level of salt, and presence of inhibitory compounds. Therefore, for each food, a series of time-temperature combinations (TTC) that are specific for the product are determined, and from this the process requirements can be predicted and compared. *D*- and *Z*-values for a number of different food products are shown in Table 3.

## 7. Pasteurized Vegetables: Degradation Kinetics for Quality Parameters

The acceptability of a food products depends upon a variety of quality parameters. The sensory attributes (appearance, color, texture, etc.) are the first criteria for acceptance or rejection of foods [37]. Thermal processing at both pasteurization temperatures and at the higher temperatures required for commercial sterility have significant effects on quality parameters particularly for sensory attributes, but the available information on the kinetic data on sensory attributes and other properties is limited, and a compilation of the available information is presented here in Table 4.

 The kinetic parameters for degradation of a food component can be calculated using one of two procedures, a steady-state procedure and a non-steady-state procedure. In a steady-state procedure, the thermal lag (heating period or come up time and postprocess cooling period) times are considered to be insignificant compared to the overall processing time, and the process is considered to occur at constant temperature. In a non-steady-state procedure, the reaction is considered to occur at a variable temperature based upon the concentration of the degraded component and secondly the temperature profile of the sample during the heating period up to the targeted process temperature along with the cooling stage is determined. Each method has its pros and cons, but for pasteurization, a non-steady-state is more appropriate because it takes into account the fact that the sample maybe subjected to various time-temperature heating profiles and that the amount of heat exposure and rate of exposure would be different throughout the heating process. Kinetic factors are determined experimentally and with the level of a targeted thermally labile component used to monitor the heating process at different time points; from this, an average retention of components can be obtained. Degradation of the component during a thermal lag is incorporated into process models.

Adams and Robertson [38] compared experimental results for the thermal inactivation of horseradish peroxidase with the predictions of the *D*- and *Z*-values and *k*- and *E*-values in reaction rate models. They found little difference between a predictive model for enzyme inactivation compared with that of microbial inactivation over a pasteurization temperature ranges, indicating that microbial survival could be predicted from a measure of residual enzyme activity.

Model thermal inactivation parameters in real foods are very difficult to process due to the complex nature of nutrient interactions and physical processes such as gelatinization that alters heat transfer properties occur during thermal processing. Commonly, a simpler model system tested under ideal conditions is used as a first approximation for mathematical modeling. A nutrient as a component in a complex food exhibits a different rate of degradation, potentially by a different mechanism, than the same nutrient in a pure state as a single component in a simple matrix such as water or buffer. For work on thermal degradation kinetics of heat labile components in vegetables, most researchers have used first-order models [39–41]. A summary of research in this area specifically for chlorophyll in vegetables is presented in Table 5 at different pH and temperature. In general, *E*_*a*_ drops with increasing pH. Quality parameters such as texture changes in legumes can be predicted using kinetic models, Table 6. For other vegetables such as asparagus, difference in *E*_*a*_ as affected by the concentration of cellulosic structural component, higher in the stem compared to the bud, was reflected in *k* and *E*_*a*_ values.

## 8. An Overview of Thermal Processes in Vegetable Processing 

### 8.1. Pasteurization

Pasteurization is a relatively mild heat treatment having the objective of inactivating pathogenic vegetative microorganisms of public health significance as previously mentioned. A mild heat treatment (70–100°C) inactivates vegetative cells and many enzymes while preserving the nutritional quality of heated vegetables. A plate-type heat exchanger (PHE) is commonly used to pasteurize low viscosity fluids (<5 Pa·sec) such as juices or milk. The cold fluid is pumped to the regeneration section of PHE followed by heating at a desired temperature (e.g., 72 to 75°C), holding for a predefined residence time (15 to 30 sec), and finally cooling to a refrigeration temperature. Viscous products can be pasteurized using a scraped-surface heat exchanger in which case the inside surface of the heat exchanger is in contact with the product. The surface is continuously scraped by molded plastic to prevent fouling. The scope of pasteurization for vegetables products is limited for shelf stable foods because vegetables tend to have higher pH [42] requiring a thermal process sufficient to inactivate *Clostridium botulinum* spores in addition to vegetative cells of pathogenic bacteria. 

Pasteurization is commonly conducted using a hot liquid medias, such as water or steam. Vegetables are packed in bags or other suitable containers and then heated in boiling water, this tends to limit the loss of flavor and soluble nutrients into the cooking water. Using steam can speed the process due to the contribution of latent heat from condensing steam that aids product heating [43]. Foods treated with moist heat (steam or hot water immersion) reduce populations of surface organisms that may be responsible for spoilage or cause illness and can also lead to enhanced refrigerated shelf life. 

### 8.2. Dielectric Pasteurization

Microwave energy for heating food product was patented in 1945 and the first commercial oven was introduced in 1955 [44]. The frequencies commonly used for microwave heating are 915 ± 25 MHz and 2450 ± 50 MHz, with penetration depth ranging from 8–22 cm at 915 MHz to 3–8 cm at 2450 MHz, depending on the moisture content of the product [45]. Dielectric heating in foods occurs due to coupling of electrical energy from an electromagnetic field within a microwave cavity with the food and then disperses this energy throughout the food product through the phenomenon of volumetric heating. Friction is created between molecules within the food resulting from dipole rotation of polar solvents and from the conductive migration of dissolved ions. The final product temperature depends upon the amount of electromagnetic energy applied, and unless the food is also processed under pressure, the product temperature does not exceed 100°C and would be insufficient to kill *Clostridium botulinum *spores. The benefit of microwave heating includes potential energy savings through reduction of processing time and higher product throughput rate. Reduced come-up time often leads to greater retention of nutrients, flavor, and texture than the same foods prepared using conductive heating processes with the same heating intensity [3, 45]. 

### 8.3. Blanching

Blanching is one of the important unit operation conducted prior to freezing, canning, or drying in which vegetables are heated up to the desired temperature for the purpose of inactivating enzymes, inducing textural changes, preserving color, flavor, and nutritional value and removing the entrapped air and metabolic gases within vegetable cells and replaces them with water, forming a semicontinuous water phase that favors a more uniform crystal growth during freezing and making the product more deformable and compressible so that it is easier to fill into containers for subsequent freezing or canning. Hot water and steam are the most commonly used heating media for blanching in the industry, but microwave and hot gas blanching have also been studied [46]. Different hot water and steam blanchers have been designed to improve product quality, increase yield, and facilitate processing of products with different thermal properties and geometries. More recently, energy conservation and waste reduction have driven further improvement of equipment design [47, 48]. Although blanching seems as a simple operation, heat transfer to a conveyed bed of product and its effects on product properties are very difficult to accurately model. Processing conditions are usually established to inactivate enzymes, but other quality parameters, such as color and texture, are commonly monitored during the process. Blanching provides either complete or partial pasteurization. For a given product, typically mass flow rate is fixed, temperature is measured, and heating media flow rate is adjusted to ensure that the temperature is kept at the set point [47].

Water blanching can involve a low-temperature long-time (LTLT) or high-temperature short-time (HTST) process. A typical temperature is ranging from 70 to 100°C depending upon the product and process conditions [20, 49] and which component, such as polyphenol oxidase is being targeted for inactivation during the blanching process. Water blanching is performed at lower temperature and results in uniform product heating but often higher leaching of minerals and vitamins [20, 49]. Some water blanchers use a screw or a chain conveyer to transport the product through a blanching tank, where hot water is added and others use a rotary drum to immerse and convey the product through the blancher. 

In steam, blanching is an alternative to water blanching. Here, product is placed on a belt conveyer that transits through a chamber containing food grade steam. It is a highly effective method since the heat transfer coefficient of condensing steam is greater than that of hot water [20, 49] and is used extensively for vegetables that are cut into small pieces. Gas blanching is based on the combustion of hot gas with steam. This type of blanching has the advantage of waste reduction and nutrient retention [48].

Microwave blanching can be conducted as a batch or continuous process. Many of the initial studies with this technology were conducted on modified home microwave ovens making comparison of unit operations that would not be appropriate at an industrial level and problematic due to variability in equipment performance. Recently, the use of fiber optic temperature probes and infrared imaging make it possible to improve process control and monitoring of microwave processes allowing firms to take advantage of the high heat penetration and efficiencies associated with volumetric heating [47]. 

Flavor, texture, and color are affected by blanching. Food quality is greatly affected by the type and extent of blanching. Mathematical equations are used to describe the effect of thermal treatments on the quality of foods. Sometimes blanching increases flavor retention and removes undesirable bitter flavor [50, 51]. However, blanching can cause undesirable softening of vegetable tissues. Calcium can be added to reduce softening [52] encouraging pectin cross-linking. A combination of low-temperature blanching along with addition of a calcium salt can be effective in firming vegetables destined for canning [20]. Blanching has both direct and indirect effects on color of vegetable by the destruction of chlorophyll and other coloring pigments and Maillard browning due the presence of reducing sugars [2, 4]. 

A number of different enzymes can cause quality problems during storage of vegetables since these remain active and cause loss of flavor and color and affect nutrient retention. Among the most problematic group enzymes are oxidative enzymes. Peroxidase (PO) is commonly the most heat resistant of these and inactivation of this enzyme is a challenge for either a pasteurization or a blanching process if product quality is to be maintained [46, 53].

## 9. Effects of Pasteurization on Quality Attributes of Vegetables

Quality is defined as the degree of compliance with technical specifications, and commonly, foods that have greater attractiveness to consumers based upon their sensory features are considered to be of higher quality. Kramer and Twigg [54] defined quality as “the composite of those characteristics that differentiate individual units of a product, and have significance in determining the degree of acceptability by the buyer.” Food quality consists of both sensory attributes that are first and immediately perceived by the human senses and some hidden quality parameters such as safety and nutritional value of the product [55]. The quality parameters for vegetables are relatively well defined although there are regional preferences with a trend in most major markets to prefer vegetables that have received less rather than more cooking. The exception to this would be markets in South Asia; although as a greater variety of cuisines are introduced into this region, a shift in preference is also anticipated. Regardless of the market, quality parameters fulfill two important aspects, one to produce a food that is safe and, secondly, to produce a consistent product that meets customer needs. 

Processed vegetables lose quality during processing and storage. The major quality attributes at jeopardy are color, aroma, taste, and texture and less tangible quality attributes include nutritional value and safety both chemical and microbial [42]. Color has a major impact on appearance, processing, and acceptability of vegetables when a vegetable is exposed to light; about 4% of incident light is reflected at the outer surface visible as specular reflectance or gloss, and the remaining 96% of the incident energy is transmitted through the surface into the cellular structure of the product, where it is scattered at small interfaces within the tissue or absorbed by cellular constituents [56]. New imagining technologies can provide accurate color measurements of fruits and vegetables using multi- or hyperspectral cameras that allow for the rapid acquisition of images at many wavelengths [8]. This kind of imagining provides information about the spatial distribution of constituents (pigments, sugar, moisture, fat, etc.) in vegetables taking advantage of the vibrational properties of important functional groups in the food, for example, 960 *η*m for water and 920 *η*m for fat in the infrared region, 325 *η*m for acetate moieties and 450 *η*m for *β*-carotene in the UV/visible region, and then mapping the relative concentrations of these components across a cut surface.

**Figure 2:**
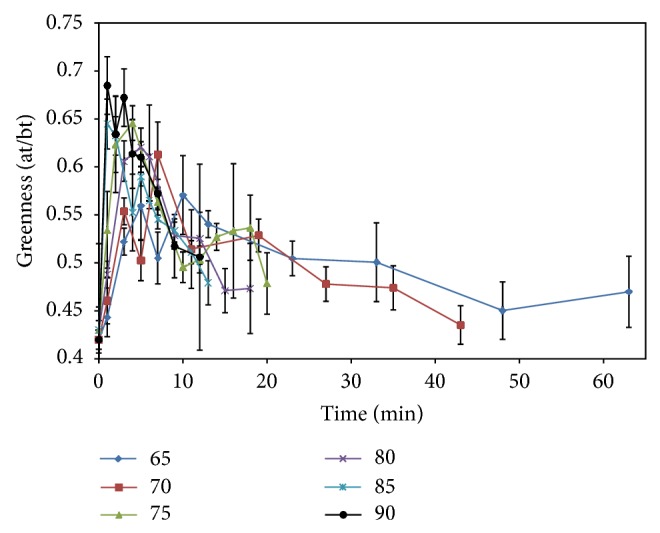
Greenness changes in whole spinach leaves (adapted from [57]).

Aamir et al. [57] reported increasing greenness during the pasteurization of spinach leaves. They reported that greenness increased during the initial heating period (1–13 min depending upon temperature). At higher temperatures, a greater increase in greenness could be observed, followed by a rapid loss at longer treatment times (Figure 2). Tijskens et al. [2] reported an increase in green color in green beans with loss of greenness upon further heat treatment. However, the chemical and physical factors associated with this change in color are not well understood. It has been demonstrated that the opacity of cells could be decreased by blanching, resulting in altering their optical properties through replacement of intercellular air with blanching water followed by the release of cellular liquids as cell membranes deteriorate [57]. In addition, in fresh produce, colorless or weakly colored green precursors that are converted into visible green components would increase color intensity during blanching treatments as chlorophyll degrades. 

The observed decrease in color later in the blanching treatment is most likely due to chemical degradation of chlorophyll [58] and a loss of the liberated colored compounds into extracellular water [16] decreasing color intensity. It was noted by Schwartz and Elbe [59] that pheophytin is only an intermediate in the thermal degradation of chlorophyll to pyropheophytin, a decarboxy-methoxylated magnesium-free chlorophyll derivative. During the heating, the central magnesium atom of the chlorophyll porphyrin ring is easily removed, thus forming pheophytin. Upon prolonged heating, pheophytin degrades further, by decarbomethoxylation of the isocyclic ring C-10 center, forming pyropheophytin derivatives, which are the final degradation products of chlorophyll [58].

Textural properties are another important factor in the quality of pasteurized vegetables. Rheology properties are measured. Rheology is the study of the deformation and flow of matter and is applied to understand the relationship between structural, mechanical properties such as tensile strength, fracturability, compression, and shear that can be related to sensory characteristics of vegetables including changes associated with thermal processing. Various researchers have defined the food texture in different ways, but the greatest insight is by Bourne [60], who clearly describes how sensory assessment of food texture can be correlated with mechanical measurements of vegetable tissue material properties.

### 9.1. Enzyme Activity as a Quality Parameter

Peroxidase (PO) is one of the most heat stable enzymes in vegetables and pasteurization processes are often designed with inactivation of this enzyme in mind since residual PO can cause off flavors during storage [61]. Thermal inactivation of PO or any other enzymes depends upon the morphological characteristics of a particular plant, the cellular structure of the edible portion, and how enzymes may be compartmentalized within vegetable cells. From a thermal processing stand point, the thickness and geometry of vegetable tissue to be processed and the thermal conductivity of the tissue will be important to the overall effectiveness of thermal inactivation. Selecting an appropriate time-temperature combination (TTC) is important for enzyme inactivation processes and maintaining the overall quality of vegetables. Usually a quick blanching process with reduced heating time maintains quality, and treatments for enzyme inactivation should take into consideration how this would affect other quality parameters. For example, Olson and Dietrich [62] found that green beans blanched in water at 100°C for 60 s retained 94.4% of chlorophyll; if blanching time increased to 300 s the chlorophyll retention was only 75.5%. First-order reaction kinetics effectively describes peroxidase inactivation and generally other enzymes such as lipoxygenase or polyphenol oxidase associated with quality loss during subsequent storage of vegetable foods. 

Peroxidase inactivation is dependent upon temperature and additives added to the blanching medium and mass/volume and dimensions of vegetable pieces. Peroxidase inactivation in Savoy beet, amaranth, and fenugreek was reduced to a negligible amount in 1 min in hot water (95 ± 3°C) followed by a potassium metabisulphite (KMS) dip [63]. This is similar to what has been observed for spinach at 85°C for 30 sec or 95°C for 15 sec [64] and at 99°C for 2 min in fenugreek leaves [65]. Okoli et al. [66] also reported that blanching of spinach and amaranth for 1 min at 95°C was sufficient for a negative peroxidase test, whereas 3–6 min steam or hot water blanching (97–99°C) is necessary for fenugreek to achieve the same effect [67]. Blanching of fenugreek leaves at 99°C for 2 min was adequate for reduction of peroxidase activity [65]. These temperatures are greater than what would be necessary for pasteurization of a product for vegetative bacterial pathogens and food borne viruses.

Microwave blanching (batch treatment, 915 MHz) of artichokes at 2 min completely inactivate PO without a loss of ascorbic acid showing advantages over boiling water at 8 min and steam blanching at 6 min which resulted in 16.7 and 28.9% loss of ascorbic acid along with peroxidase inactivation [68]. There is some evidence that the quality of blanched or processed food is superior even if some peroxidase activity remains because the additional time for complete inactivation can result in browning, excessive textural softening, or changes in appearance such as ragged edges. The percentage of residual activity that can remain without causing adverse quality changes varies from product to product for peas (2–6.3%), green beans (0.7–3.2%), cauliflower (2.9–8.2%), and brussels sprouts (7.5–11.5%). Another problem associated with the complete inactivation of peroxidase is the presence of 1–10% of more heat-stable peroxidase isoenzymes in most vegetables [51, 69], which are difficult to inactivate. In some vegetables, complete inactivation of peroxidase enhances nutrients loss [70]. 

The thermal sensitivity of an enzyme is affected by a number of different factors such as pH with a maximum stability observed at mild acidic conditions [71]. For example, peroxidase in asparagus was more stable at pH 6.0 and less at both higher and lower pH over a range from 4 to 7 [72]. 

Lipoxygenase is extensively found in vegetables and is often involved in off-flavor development and color loss [73]. Several researchers suggested that analysis of lipoxygenase activity may be a more accurate index of blanching adequacy instead of peroxidase [73, 74], even though inactivation of lipoxygenase requires less heat treatment. A time-temperature combination based upon an 80% reduction of lipoxygenase in carrots follows a first-order kinetic model [75] and may be sufficient inactivation for a blanching process. Thermal stability of lipoxygenase is consistent for pH in the range of 4–7 at least for asparagus [76]. 

Polyphenol oxidases are another class of enzymes important for food quality and catalyzes the oxidation of phenolic compounds to produce brown pigments following tissue damage and exposure of cut surfaces of fruits and vegetables. Browning is accelerated at higher temperatures and also at neutral pH. The thermal stability of PPO is high at neutral pH and, in mushroom, constant from a pH of 5.5–7.5 [77]. Browning leads the development of off-flavors and losses in nutritional and overall sensory quality and is a problem for sliced fresh, frozen, and dehydrated items. Many plant materials have one or more isozymes of PPO, some of which are highly thermostable. PPO has been used as blanching indicator for potatoes, apples, peaches, mango, banana, and other products. Following PPO initiation of phenols, hydroxylation at the o-position adjacent to an existing OH group occurs with oxidation to o-benzoquinones and then nonenzymatic polymerization proceeds to form melanins [78]. PPO actively often correlates to color changes making color measurement a suitable indirect index of PPO activity [78]. 

### 9.2. Effect of Pasteurization and Commercial Sterilization on Vegetable Color and Pigment Content

The color of foods is one of the most important quality factors for vegetables and plays a considerable role in the overall acceptability of foods. Color is a component of total appearance and incorporates visual recognition and assessment of the surface and subsurface properties [79–81]. Instrumental color measurement provides an indication of visual quality and tristimulus colorimetry is a well-established rapid and simple instrumental method to predict the visual perception of foods [82, 83]. 

Color by tristimulus colorimetry is commonly represented in terms of *L*, *a*, and *b* values (brightness, green to red, blue to yellow, resp.) or a combination of these three parameters depending upon the nature of the pigment in the food material and the optical properties of the food surface [2, 80, 81, 83–87]. Parameters derived from *L*, *a*, and *b* values, such as the total color change (Δ*E*), are commonly calculated. Chroma indicates color saturation and is proportional to color intensity. Hue angle is frequently used to specify color in food products with an angle of 0 or 360° representing red hue, and angles of 90, 180, and 270° indicating yellow, green, and blue hues, respectively. Browning index (BI) is a parameter associated with heating processes involving enzymes or oxidative browning [2, 80, 81] and is calculated from *L*, *a*, and *b* values. 

One of the most important parameters in quality assessment for vegetables is a quantitative assessment of greenness. This reflects changes to chlorophyll that occur during cooking and commercial sterilization processes [88]. For pasteurization, an increase in green color has been observed during the initial stage of heating in broccoli (40–96°C, 180–4 min) [2, 79, 83, 84] and as an increase in green color observed during the blanching of spinach and mustard greens at the ranges of 75 to 115°C and 50 to 120°C, respectively. Heat induced color changes (from bright green to olive brown) are attributed to the conversion of chlorophylls *a* and *b* to their respective pheophytins and further degradation to pyropheophytins [2, 87]. Upon prolonged heating, pheophytins is formed by exchange of Mg^2+^ with H^+^ in the center of the porphyrin ring of chlorophyll [2]. Others have shown that instrumental color measurements compare well with chemical determination of chlorophyll loss in heated green vegetables such as broccoli and shows the advantage of greenness as a useful quality assessment measure that closely reflects consumer perceptions since green color and visual appearance are more important for preference than residual chlorophyll content [58]. 

Thermally induced loss of quality, including color, can be predicted from kinetic models, usually first order. A number of different and useful models have been developed for pigment and color degradation in fruits and vegetables such as broccoli [2, 58], peas [87, 89], leafy green vegetables [59, 79–81, 84], chili [82, 83], and peach [90]. 

Studies on kinetics of color change in green vegetables are commonly conducted with macerated tissue or purees to remove some of the confounding factors associated with biological variability. For example, visual changes in green color and the kinetics of color change in spinach puree under different temperature treatments, such as 50 to 100°C for 20–60 min in studies by [79, 84], and from 75 to 115°C for up to 20 min [82, 83], showed a predictable and consistent loss in color. Few studies have been conducted on whole tissues. In one study, using whole tissues studied changes in color and moisture diffusivity in whole leaf spinach and okra during microwave dehydration [80, 81]. 

Most studies on color change from heat treatments refer to a decrease of green color [2], but only a few researchers mention an initial increase in green color upon heating. For example, [91] noticed an increase in green color of green asparagus during the initial stages of heating between 70 and 98°C; [2] also reported a change in green color due to the heat treatments consisting initially of an increase in color followed by a decrease in greenness in both broccoli and green beans (40 to 96°C). Failure to detect this phenomenon in earlier studies may have been due in part to the time points selected for monitoring the heat treatment. Most studies focus on prolonged heating at higher temperatures, for example, under sterilization conditions and not at the time-temperature combinations for pasteurization conditions where this phenomenon is apparent. In many studies, an increase in green color is not observed because vegetables were blanched before color measurements were performed. A summary of color loss associated with heating in green vegetables, carrots, and tomato is presented in Table 7. 

### 9.3. Effects of Thermal Processing on Vegetable Texture

Textural changes occur in food during thermal processing, and they cause softening of the tissues due to physical and chemical changes that may render the food unacceptable to consumers [35]. Understanding the kinetics of textural degradation can be used to optimize a thermal process minimizing textural degradation and yielding a higher quality product. A number of studies report thermal softening and textural degradation kinetics for vegetables. Textural degradation kinetics is a very complex phenomenon and various approaches have been used to analyze the data of textural degradation during thermal processing of vegetables. The literature indicates that thermal degradation kinetic of vegetables follow first-order reaction kinetics. For example, a kinetic model for green pea softening at 110°C expressed as firmness fit empirical data [92]. Kinetic data on heat induced textural changes is available on a variety of vegetables [33, 93] with a summary of data for softening presented in Tables 7 and 8.

This model has been successfully applied to asparagus, peas, knoll-kohl, and carrots [33, 46]. Softening for fruits and vegetables occurs in the cell wall and middle lamella components, and a two-phase model can reflect these changes in the tough and fibrous cell walls, present in appreciable amounts and component in the Bourne model [92]. The cell wall is made up of cellulose fibrils imbedded in a matrix consisting of pectin substances, hemicellulose, proteins, lignin, lower molecular weight solutes, and water. Cellulose gives rigidity and resistance of tearing, while pectin and hemicellulose grant plasticity and ability to stretch. 

The mechanism for textural changes during thermal processing may be different at high temperature than at lower temperature [93]; however, this two-stage model is robust and should provide a basis for developing new pasteurization processes and verification protocols as required under newly mandated hazard analysis critical control point (HACCP) based food protection programs. 

The main defects in many studies of softening caused during heating have been the lack of correction of thermal lag and uneven heating of samples, with loss of turgor and changes in cell wall polysaccharide matrix [93] in thermal processing models. A thermal lag correction procedure was proposed by monitoring the temperature history at the geometric center of a cylindrical container showing that this strategy could be used to estimate the transient temperature distribution [94, 95]. The lag correction method has been employed for softening studies by others [25, 39, 96] and this method is easy to use. Results are comparable to more elaborate schemes such as numerical integration of the transient heat conduction [97]. 

Two-step mechanisms for texture degradation have some limitations, particularly at the second step where the degradation reaches equilibrium in the softening stage and the calculated activation energy becomes negative. Because of this, a fractional conversion technique has been employed to describe thermal softening of vegetables inferring that first-order reaction kinetics were appropriate to describe texture degradation during thermal processing [23, 98, 99]. 

Rizvi and Tong [98] applied the fractional conversion technique taking into account nonzero equilibrium texture properties. The texture index expressed as the extent of texture change, *f*, at any time, *t*, is expressed as follows:

(9) $$\begin{array}{c} f=\frac{\left(TP_{0}-TP_{t}\right)}{\left(TP_{0}-TP_{\infty }\right)}, \end{array}$$

where *TP*_0_ is the initial texture property at zero time, *TP*_*t*_ is the texture property at a given time, *t*, and *TP*_*∞*_ is the nonzero equilibrium texture property after prolonged heating time.

According to Levenspiel [100], for 1st order reaction kinetics, (1 − *f*) plotted against time (*t*) is linear, and the rate constant (*k*) is the negative of the slope. The equation is written as

(10) $$\begin{array}{c} \ln\left(1-f\right)=\ln\frac{\left(TP-TP_{\infty }\right)}{\left(TP_{0}-TP_{\infty }\right)}=-kt. \end{array}$$

When we predict the texture index as a function of heating time (*t*) at a constant temperature, (10) can be rearranged as follows:

(11) $$\begin{array}{c} TP_{t}=TP_{\infty }+\left(TP_{0}-TP_{\infty }\right)\cdot \exp\left(-k\cdot t\right). \end{array}$$

The main benefit of applying the fractional conversion technique in data reduction is that there is no need to standardize the experimental protocol [99]. Process activation energies during thermal softening of potato and texture degradation of carrot varied significantly. Some variations in texture measurements were explained based upon differences in sample size [92, 101–104]. Mittal [104] concluded that textural properties such as hardness, brittleness, cohesiveness, elasticity, gumminess, chewiness, and penetration force decreased with the increase in potato and carrot treatment temperature (20–90°C) showing the effects of thermal processing on textural softening from the breakdown of the cellular material. Most vegetables soften when heated due to the loss of turgor and changes in cell wall polysaccharides and lignified materials. The edible portions of vegetables are composed of parenchymatous tissue and the cells of this particular tissue are thin walled and are joined together through pectin substances of the middle lamella. Another mechanism for softening of the cell wall involves decreased cohesiveness of the matrix and intercellular adhesion, which is followed by absorption of water by exposed polysaccharide moieties. Studies of potato hardness showed significant changes up to 60°C but fewer changes after 70°C due starch swelling and gelatinization at temperature around 60–70°C [105]. In a case of carrot, textural changes are promoted due to the de-esterification of the pectic substances, either by formation of gel-like structures of the pectinic acid produced by the enzyme or reaction of the free carboxyl groups with divalent ions [104]. The stress relaxation data for carrot has been described in a linear Maxwell model as follows:

(12) $$\begin{array}{c} E\left(t\right)=E_{0}+\sum E_{i}\exp\left(-t/\tau_{i}\right), \end{array}$$

where *E*(*t*), *E*_0_, and *E*_*i*_ are the decaying parameters (force or stress), *τ*_*i*_ is the relaxation time, and *t* is for time [104]. 

Blanching affects the texture of vegetables either positively or negatively. For some vegetable, texture softening is desirable, but for others, it is undesirable, such as sweet potato, carrot, and jalapeno pepper. The low-temperature long-time (LTLT) blanching has been found to be effective for snap beans and cauliflower, tomato, potato, and carrot [20, 106–109]. Blanching at 55 to 85°C for times ranging from several minutes to several hours showed a firming effect for some vegetables. For sweet potato, blanching at 62°C for 90 min resulted in maximum firmness, while high-temperature blanching disrupted cell integrity and cell adhesion and reduced tissue rigidity [108]. Blanching at 55°C for 60 minutes produced maximum firmness of jalapeno [106]. The LTLT blanching followed by canning of vegetables caused more fracturability and hardness of texture and also resulted in gumminess and springiness of canned sweet potato, more firmer texture in carrot, jalapeno pepper, and green beans [20, 106–109]. During LTLT blanching, pectinase enzymes partially demethylate pectin leaving OH sites free on the pectin chain to cross link with other pectin molecules through a calcium bridges resulting in a firmer texture [38, 110]. 

Thermal textural softening of vegetables is caused by many factors like hydrolysis of pectin, gelatinization of starches, and solubilization of hemicelluloses and loss of cell turgor resulting in changes in the cell wall, particularly the middle lamella [20, 108].

In some cases, high-temperature short-time (HTST) blanching has many advantages over low-temperature long-time (LTLT) blanching. Carrot tissues subjected to HTST (100°C for 0.58 min) retained a firmer texture than if subjected to LTLT (70°C for 71.10 min) [75]. Two-stage blanching was found to be more effective as compared to single-stage blanching. In two-stage blanching, first blanch is at 70°C followed by high-temperature blanching resulting in firmer texture of green beans [20]. 

## 10. Effect of Thermal Processing on Sensory and Nutritional Quality of Vegetables

Vegetables are a primary source of macronutrients including fiber and carbohydrates and micronutrient vitamins, minerals, polyphenolics, carotenoids, and glucosinolates. The most important of them are dietary fiber, folate, potassium, vitamin A, and vitamin C [111]. Consumers prefer vegetables that are a good source of dietary fiber and many vitamins and minerals but unfortunately are unable to distinguish between the vegetable foods that have high versus low concentrations of phytonutrients. 

Among different vitamins in vegetables, ascorbic acid (vitamin C) is the most heat labile and easily oxidizes by ascorbic acid oxidase. The loss of ascorbic acid is increased with increasing temperature and time during heat treatments and with blanching; loss is affected by the style of blanching since ascorbic acid leaches out easily [112]. With respect to ascorbic acid retention in potato, significant differences were observed during blanching at 80 to 93°C [113]. Microwave blanching may result in greater retention than steam blanching as observed in a study of broccoli [114, 115]. However, this same effect was not found comparing microwave and a boiling water treatment of beans prior to freezing. Comprehensive data on vitamin C retention can be found for fruit juices with little data available for vegetable products, although the trends would be similar. As an example, comparison of vitamin C loss during heat treatments is presented in Table 9 for grape and Table 10 for orange juice.

Nutrient leaching is dependent upon changes in cell morphology during heating. Cell walls are stiff and give structural integrity to the plant cell, but the cell membranes are flexible. However, during heating in a moist environment, the cell absorbs water; the cell membrane presses against the cell wall. This is called turgor pressure and it makes vegetable crispy [63]. A loss of water causes vacuoles to shrivel and cell membrane to pull away from the cell wall allowing vitamin C and other water soluble nutrients inside the plant cell to escape [63]. Steam blanching effects the nutritional quality of many vegetables such as maximum loss (30%) were observed in broccoli, 14% in carrot, while green beans showed the least effect as compared to microwave blanching [116]. Steam blanching as a pretreatment, though causing some initial losses of carotene due to degradation of tissues, can yield higher overall carotene retention during dehydration and subsequent storage [22].

Addition of sulfite agents following blanching can reduce leaching in some cases resulting in greater retention of *β*-carotene, ascorbic acid and chlorophyll in amaranth, and fenugreek in hot water (95 ± 3°C for 1 min) followed by a potassium metabisulfite (KMS) (5 g/L in water) dip for 1 min. Losses are dependent upon the type of vegetable treated with high leaching observed in Savoy beet, 53% of carotene and 80% ascorbic acid (dry weight basis) as compared to fenugreek and amaranth [63]. Song et al. [117] concluded that blanching of vegetable soybean at 80°C for 30 min, 90°C for 20 min, and 100°C for 10 min led to significant decreases in glucose, fructose, and sucrose due leaching but observed little loss of amino acids and tri- or tetrasaccharides and vitamin B1. This is because only 19% of the amino acids in vegetable soybean are present in a soluble form. 

High-temperature short-time (HTST) blanching is beneficial keeping in view the nutritional value of vegetables. Blanching of edamame (young soybean or vegetable soybean) at different time-temperature combinations (TTCs) (80°C for 30 min, 90°C for 20 min) resulted in nutrient losses such as sugar and vitamins B_1_, B_2_, and C, the loss was lowest at 100°C for 10 min [115]. 

## 11. Thermal Processing and Effect on Pigment and Bioactive Compounds 

Anthocyanins are the bioactive compounds present in different fruits and vegetables and are the basis for the red, blue, and purple colors of fruits and vegetables. They have a series of conjugated bonds capable of absorbing a light up to 500 nm, which provide basis for the red, blue, and purple colors in different fruits and vegetables. They are readily degraded during thermal processing leading to loss of color and nutritional quality.

**Figure 3:**
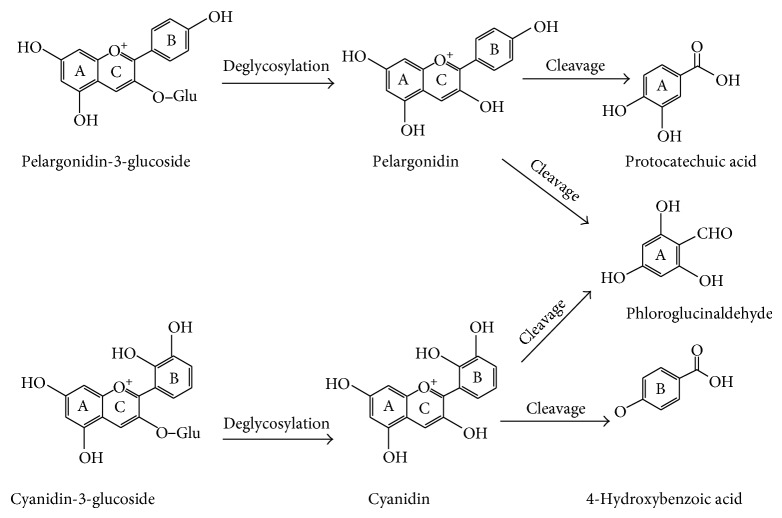
Thermal degradation mechanism of two common anthocyanins (adapted from [121]).

Anthocyanins are glycosylated anthocyanidins; sugars are attached to the 3-hydroxyl position of the anthocyanidins [118] (Figure 3). Various structural modifications including the number of hydroxyl groups, degree of methylation, nature and number of sugar moiety, and nature and number of aliphatic or aromatic acids [118, 119] result in the creation of a number of anthocyanin compounds in foods. Anthocyanin degradation results from oxidation during thermal processing and cleavage of covalent bonds with the degree of degradation depending upon the severity of the heat treatment. Degradation rate of anthocyanins increases during processing and storage as temperature rises [120]. 

Anthocyanins are rapidly degraded during thermal processing even at pasteurization temperatures [121, 122]. The stability of anthocyanins depend upon many factors besides heat such as pH, storage temperature, chemical structure of the anthocyanin compound, presence of UV light, oxygen, oxidative and hydrolytic enzymes, proteins and phenolic compounds that could have a protective effect, and the metallic ions that could enhance oxidation. The exact mechanism for the stability of anthocyanins is difficult to establish, but the phenolic acids such as ferulic and syringic acids play a role in stability [1]. Magnitude and time of heating have a strong influence on anthocyanin stability, and after 3 h of heating at 95°C, only 50% of anthocyanin based pigments in elderberry were retained [123]. This level of loss is fairly typical for heating processes. Storage temperature plays critical role for anthocyanin loss and this will play an important in preserving remaining anthocyanins following pasteurization [124], since retention is greatly enhanced as storage temperature is lowered. Fast degradation of anthocyanins in colored juices and nectars was shown when stored at 37°C compared to refrigerated storage [125, 126]. Much slower degradation is also observed at 20°C compared to 37°C [127]. 

Blanching, boiling, and steaming resulted in anthocyanin losses of 59%, 41%, and 29%, respectively, in red cabbage [128]. However, the anthocyanins in certain products appear to be more stable. For black carrot, anthocyanins showed reasonable stability during heating at 70–80°C [125, 126] and 70–90°C [129] with these differences in stability related to anthocyanin structure and the pH value [128]. Black carrot anthocyanins may be more stable to heat and pH as compared to other sources due to the presence of di-acylation in anthocyanin structure. Acylation of the molecule is believed to improve anthocyanin stability by protecting it from hydration [125, 130]. The presence of inter- and intramolecular copigmentation with other moieties, polyglycosylated, and polyacylated anthocyanins provides greater stability towards change in temperature, pH, and light [131, 132]. Storage temperature also had a very strong influence on the stability of black carrot anthocyanins colored juices and nectars. 

 Dyrby et al. [133] reported greater stability of anthocyanins present in red cabbage at temperature ranges from 20 to 80°C and at treatment times ranging from 15 to 360 min as compared to anthocyanins in black currant, grape skin, and elderberry in a soft drink model system. This was thought to be due to the protection of flavylium system through copigmentation in cabbage. 

The most stable anthocyanins during storage at 8°C for 12 months were cyanidin and delphinidin-ruti-nosides, and storage at 4°C in an inert atmosphere may induce a slow degradation process of anthocyanins. In beverages, reactions of sugar and ascorbic acid may enhance transformation at anthocyanins to brown compounds [134]. 

Oxygen plays an important role and accelerates the degradation of anthocyanins either through a direct oxidative mechanism or through the action of oxidizing enzymes [124]. Enzymes such as PPO catalyze the oxidation of chlorogenic acid (CG) into the corresponding O-quinone in the presence of oxygen. These quinones further react with anthocyanins to from brown condensation products [135]. Anthocyanin degradation under isothermal heating is reported to follow the first-order reaction kinetics for different fruits and vegetables [125, 136–138]. Degradation kinetics of anthocyanins can be modeled as follows [121]:

(13) $$\begin{array}{c} C_{t}=C_{0}\times \exp\left(-kt\right)\\ T_{1/2}=\frac{\log e^{2}}{K}\\ \text{Log}\left(\frac{K_{T}}{K_{0}}\right)=-\frac{E_{a}}{2.303\times R}\left[\frac{1}{T_{1}}-\frac{1}{T_{2}}\right], \end{array}$$

where *C*_*t*_ is anthocyanin concentration (mg/100 mL) at time *t* (min), *C*_0_ is the initial concentration (*t* = 0), *K* is a rate constant (min^−1^), *E*_*a*_ is activation energy (KJ mol^−1^), and *R* is universal gas constant (8.314 KJ mol^−1^ C^−1^).

**Figure 4:**
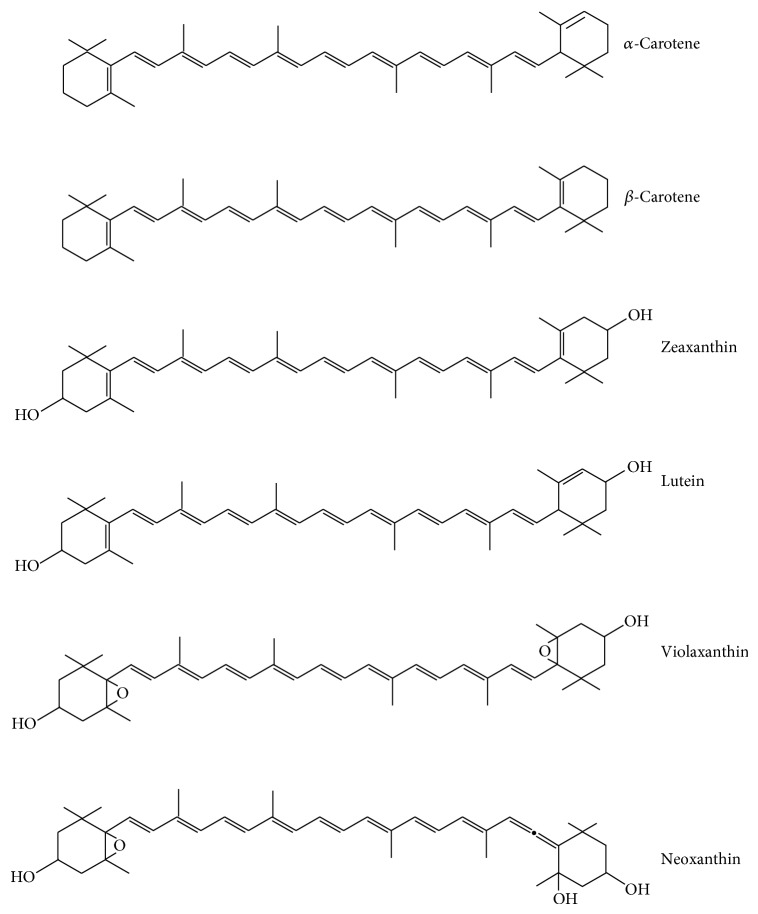
Chemical structure of the main chloroplast carotenoids of higher plants (adapted from [140]).

Carotenoids are also a class of important pigments and micronutrients in the human diet and a group of naturally occurring fat-soluble pigments. Carotenoids are abundant in yellow, orange, and dark green leafy vegetables. The degradation of carotenoids is a major issue in vegetables. Carotenoids are comprised of eight isoprenoid units (Figure 4). They are classified on the basis of chemical structure as oxycarotenoids or xanthophylls. The primary carotenoids required by plants for photosynthesis are *β*-carotene, violaxanthin, and neoxanthin. Other carotenoids localized in fruits and flowers include *α*-carotene, *β*-cryptoxanthin, zeaxanthin, antheraxanthin, capsanthin, and capsorubin [139]. Carotenoids play a beneficial role in different foods as colorant, precursors of vitamin A, and as antioxidants [140]. Carotenoids may have important plant protective roles; for example, corn carotenoids modify enzymes activity inhibiting synthesis of aflatoxin by *Aspergillus flavus* by up to 90% and *Aspergillus parasiticus* by 30% [141].

Unfortunately, carotenoids are unstable under a number of different processing and storage conditions including heating [142]. *β*-Carotene loss is accelerated in the presence of high levels of linolenic acid, and some components of pepper enhance *β*-carotene oxidation. Ascorbic acid can serve as either a prooxidant or antioxidant for *β*-carotene depending upon its concentration and the presence of copper ions. The presence of enzymes such as peroxidase may have a confounding effect. A high ascorbic acid concentration (100 *μ*mol/g of cellulose) in the presence of copper ion inhibits the prooxidative activity of peroxidase [143].

Moderate thermal processing does not cause a loss of many important carotenoids unless oxygen or ultraviolet light are present or heating is extensive. One hour boiling of tomatoes leads to total destruction of epoxycarotenoids [144]. Similar results were obtained with other vegetables from carotenoids saponification resulting in greater loss of xanthophylls compared to carotenes [145]. Slower drying, likely at lower temperatures, appears to result in lower loss of carotenoids in peppers [146, 147]. During milling of pepper, the carotenoids most affected were *β*-carotene followed by *β*-cryptoxanthin and zeaxanthin, while the most stable were capsanthin and capsorubin, unfortunately no additional information was provided as to the stability of these components following exposure to moist heat treatments. 

Chen et al. [148] studied the effect of processing on the carotenoids content of carrot juice comparing pasteurization at 105°C for 25 seconds with 110°C for 30 seconds finding minimal loss of *α*-carotene but up to 45% for *β*-carotene and 30% for lutein. Sterilization processes at 121°C for 30 min resulted in higher losses of 55.7% for *β*-carotene, 60% for *α*-carotene, and 50% for lutein [149] with indications that losses of carotenoids would be minimal under pasteurization conditions.

Blanching (98 ± 1°C for 5 min), cooking (98°C for 15, 30, and 60 min), and drying using direct sunshine and shade (photo protected) at ambient temperature (25°C ± 6°C for 24 ± 12 hours) of regionally important Tanzanian vegetables (amaranth, cowpea, peanut, pumpkin, and sweet potato leaves) [150]. A blanching treatment resulted in a reduction of *β*-carotene concentration, significant increase in *α*-carotene concentration, an apparent increase in recoverable carotenoids as a result of cooking, and reduced the concentration of carotenoids from sun drying [150]. Thermal processing increased the vitamin A activity of all the vegetables tested in this study except amaranth. Thus, blanching and cooking may be beneficial processes for increasing the nutritional availability of provitamin A. For a country such as Tanzania where vitamin A deficiency is common, consumption of 100 g of dry weight of these vegetables cover the recommended daily intake for vitamin A for both children and adults [150]. In a similar study, blanching, sautéing (cooking in hydrogenated fat), and sun drying of the leaves of bathua (*Chenopodium album*) and fenugreek (*Trigonella foenum graecum*) reduced carotenoid content [151]. Sautéing resulted in greater retention of carotenoids suggesting a protective effect for fat at least when shorter time lower temperature processes are used. Blanching for a short time (5 min) and cooking in a pressure cooker resulted in high retention of carotene [151], but dehydration reduced carotenoid content. 

Kinetic studies of carotenoid degradation are usually characterized as first-order reactions; for example, [152] studied spinach and carrot photostability and found that carotenoid degradation was first order with the carotenoids in carrots being more stable than those in spinach. In carrot juice, photodegradation and photoisomerization reactions were slower than anticipated indicating that protective factors may be present in juice [153]. Lutein tends to be highly stable and violaxanthin the least stable carotenoid. References [148, 149] concluded that when carrot juice was acidified, pasteurized, and then subjected to light or dark storage at different temperatures (4, 25, and 35°C) for 3 months, it showed higher degradation of lutein at 35°C.

**Figure 5:**
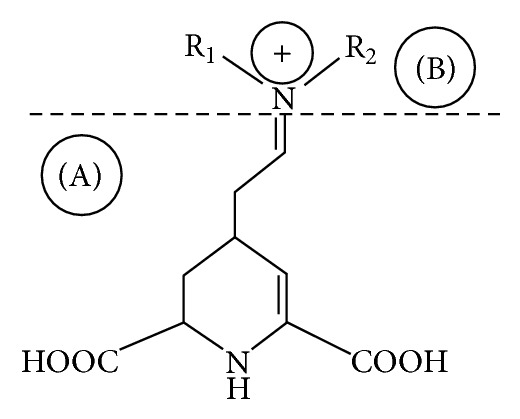
Betalains general formulae. (A) Betalamic acid moiety is present in all betalain molecules. (B) The structure will represent a betacyanin or a betaxanthin, depending on the identity of the R_1_ and R_2_ residues (adapted from [154]).

Betalains are bioactive compounds commonly found in red and yellow vegetables. There has been a great deal of interest in using these compounds as well as natural extracts of vegetable such as beets as natural colorants or to increase the quantity of bioactive components in foods. Betalains like anthocyanins and carotenoids are generally lost during food processing operations and strategies to retain them and remain an important consideration for the food industry. The names of these compounds are derived from the Latin word for red beet (*beta*) and Greek terms *xanthos* for yellow and *kyanos* (blue color) for the red-purple betacyanins. Color is dependent upon the R_1_-N-R_2_ moieties [154] (Figure 5). 

Betalains were originally called as caryophyllinenroth and after that they were renamed as rubenroth and chromoalkaloids. Betalains are the immonium derivatives of betalamic acid and their chromophore can be described as a protonated 1, 2, 4, 7, 7-pentasubstitued 1, 7-diazaheptamethin system [155]. Betacyanins consist of betanidin as basic structural unit, and its C15 epimer, isobetanidin [156]. Betaxanthin are comprised of different proteinogenic and nonproteinogenic amino acids and biogenic amine-conjugated moieties of betalamic acids [156].

Stability of betanin solutions is pH dependent, and optimal pH for maximum betanin stability in the presence of oxygen is in the range from 5.5 to 5.8. Red beet solutions had maximum stability at pH 5.5, the normal pH for beets [157, 158]. Temperature affects the stability of betanin and its thermostability is pH dependent and partially reversible. Thermal degradation of betanin follows first-order reaction kinetics [157–159]. Thermal degradation of betalains produced activation energies in the range of 17–21 Kcal-mol^−1^ for the forward reaction and 0.6 to 3.5 for reverse reaction; [92, 157, 159, 160] concluded that the rate of betanin degradation increased 15.6% after pigment was exposed to daylight at 15°C and that degradation was a first-order reaction. In the presence of fluorescent light, degradation was higher at pH 3.0 as compared to pH 5.0. 

The greatest stability of betalains has been reported in foods of low moisture and Aw with degradation also being first order. *Aw* has a pronounced exponential effect on pigment stability. Pigment stability decreases in one order of magnitude when *Aw* was increased from 0.32 to 0.75 [101, 161]. Oxygen causes product darkening and loss of color. Reference [159] stored buffered betanin solutions at pH 7 under atmosphere of air and nitrogen for 6 days at 15°C; it was observed that color degradation increased up to 15% due to oxygen exposure.

Beet roots represent the main commercial source of betalains in the form of concentrates or powders [159, 162, 163]. Many preharvest and postharvest factors and processing conditions can influence the recovery of these natural beet colorants and the bioactivity of recovered antioxidant components in addition to the betalains in beets. The average pigment content of beets is approximately 130 mg/100 g fresh weight, but newer red beet varieties produce around 450 to 500 mg/100 g fresh weight [159, 163, 164].

**Figure 6:**
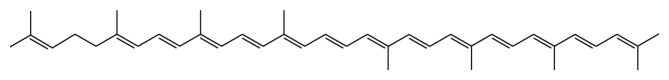
Molecular structure of lycopene (adapted from [169]).

Lycopene is a bioactive carotenoids present in many fruits and vegetables (Figure 6). It serves as a precursor for vitamin A.  Table 11 provides data on the lycopene content of different fruits and vegetables. Lycopene is a naturally occurring fat-soluble pigment present in some plants and microorganisms. It serves as a light gathering pigment and protects these organisms against the toxic effects of oxygen and light. It is the main carotenoid responsible for the red color and the beneficial effect of different fruits and vegetables [165]. In some cases, the color of lycopene is enclosed by the green chlorophyllic pigments in green vegetables and when the chlorophyll content decreases as plant mature, leaving the lycopene and other carotenoids responsible for the bright colors of different fruits and vegetables [165]. Lycopene functions as antioxidant and exhibits a high physical quenching rate for singlet oxygen in vitro. The quenching constant is more than double that of *β*-carotene and 10 times than that of *α*-tocopherol. Lycopene plays an important role as a micronutrient and may provide a protective effect against prostate, lung, and other cancers [165–167]. During processing and storage, lycopene bioactivity depends on the total lycopene contents and extent of isomerization. Major causes of lycopene isomerization are heat, light, and acid [168]. Nutritional quality and health benefits of lycopene depend not only on the total lycopene content but also on the formation of lycopene isomers. Characterization and quantification of lycopene isomers would provide a better understanding of the potential nutritional quality and health benefits of the processed tomato products [169, 170]. 

Lycopene is an important natural color ingredient in different food formulations, and the use of tomato paste as a colorant makes this commercially important natural pigment. Lycopene undergoes degradation through isomerization and oxidation during thermal processing which affect the sensory and nutrition quality of the fruits and vegetables [170]. It is found primarily in tomatoes, tomato products, and other fruits and vegetables and is an important part of western diet and the most abundant in human serum [144, 165]. It is the main carotenoids responsible for the red color of tomato products and has been suggested as the main phytochemical responsible for the beneficial effects of tomatoes [165]. 

Lycopene is restricted to the chloroplasts of fruits and vegetables and found among the thylakoid membranes in the photosynthetic pigment-protein complex [171, 172]. In fruit and vegetable, the dominant pigment in the chloroplasts is green chlorophyll, but when it degrades, the color changes from green to white, and further when chlorophyll in the chloroplasts is reduced, lycopene is biosynthesized with changes in the ultrastructure and color changes from white to red [173, 174]. The final stage of chromoplast development is the formation of lycopene crystals [175].

Heating of tomato juice at 90–100°C for 7 min resulted in a 1.1–1.7% decrease in lycopene, but at higher temperature loss is even more such as 17.1% at 130°C for 7 min [176]. The nature and extent of lycopene degradation depends upon temperature and time of heating. In solution, 26.1% of the lycopene was lost when heated at 65°C for 3 h and 35% at 100°C for 3 h [176]. 

## 12. Conclusion: Quality Optimization for Vegetables

Quality optimization for thermal processing of vegetables involves balancing retention of quality attributes and inactivation or destruction of pathogenic and spoilage microorganisms and enzymes. The amount of heat that a product receives depends upon its consistency, thermal processing conditions, and the package size, type, and configuration [36]. The need to optimize thermal processing conditions is necessary to maintain quality and usually the rate of a chemical reaction will double with each 10°C rise in temperature while bacterial destruction may increase by as much as a factor of ten [36]. 

Exposure of vegetables to higher temperatures initially disrupts essential metabolic and respiratory processes. Following this, cell structure changes occur, therefore, knowledge of cell structure and how this causes loss of texture and leaching of nutrients as a result of thermal processing. Increased fluidity of membrane lipids at higher temperatures (above 50°C) correlates with the loss of functional cell compartmentalisation considerably enhancing membrane permeability [177], leakage of electrolytes, and reduction of turgor pressure. Temperature induced phase transitions in lipid causes alterations in membrane structures leading to transmembrane protein conformational changes. Heat stress causes many cell proteins to unfold, aggregate, and precipitate [178]. Lipid peroxidation is one induced by heat shock and can also result in various structural and functional disturbances in the plant cell [179]. Understanding and controlling these processes will allow for improvement of shelf life and quality of processed vegetables, preferably with technologies that can maintain greater “fresh-like characteristics.” Quantification of the degree of cellular disruption will allow researchers to compare and optimize preservation processes [21].

The food industry particularly the vegetable processing industry has faced many challenges for the production of thermally processed foods that fulfill all the safety and quality demands of the consumers. Thermal processing remains the most common technique for the production of processed vegetables that are free from food-borne pathogens. Reducing detrimental changes that affect quality parameters such as color, texture, and flavor, sensory and nutritional value must be taken in to consideration. The quality of vegetables is based on four main attributes such as color, texture, flavor, and nutritional value which are mainly affected by thermal processing. The knowledge of chemical and biochemical nature of different pigment present in vegetables like chlorophyll, carotenoids, lycopene, betalains, and xanthophylls among others, and the loss of color or bioactive properties is becoming more important as consumer demands greater levels of these newly discovered micronutrients in their foods.

The efficiency of thermal processes such as pasteurization and the resultant product quality is dependent upon the time-temperature combination (TTCs) used. The most appropriate time-temperature combination will depend upon the morphological characteristics, chemical composition, and heat transfer properties of the vegetable tissue. Kinetic models have been developed to evaluate quality changes that occur during thermal processing and are commonly used to select and appropriate subset of possible TTCs for processing vegetables with empirical validation of these conditions to find those that provide the greatest retained quality and highest product safety. 

## Figures and Tables

**Table 1:** pH values of selected vegetables.

Vegetable	pH
Rhubarb	3.1–3.4
Tomatoes	3.9–4.5
Eggplant	4.7–5.7
Cauliflower	4.9–5.5
Potatoes	5.0–6.0
Cabbage	5.2–6.3
Broccoli	5.2–6.5
Artichoke	5.38–6.89
Asparagus	5.5–5.8
Celery	5.5–6.0
Pumpkin	5.5–7.5
Cucumber	5.6
Endive	5.8
Bean	6.0
Parsnip	6.0
Zucchini	6.0
Coriander leaf	6.0–6.25
Lettuce	6.0–6.4
Beets	6.0–6.5
Turnip	6.0–6.5
Jalapeno pepper	6.0–6.6
Onion	6.0–6.7
Spinach	6.0–7.0
Radish	6.0–7.0
Peas	6.0–7.0
Carrot	6.3
Okra	6.5
Brussels sprout	6.5
Leek	6.5–7.0

Data from [4, 18, 42, 82, 83, 180, 181].

**Table 2:** Kinetic parameters associated with quality attributes of vegetables.

Reaction type	Vegetable type	Order of reaction	Temperature range (°C)	Activation energy (KJ/mol)	Reference
Thermal inactivation of peroxidase and lipoxygenase	Broccoli (floret)	Biphasic 1st	70–95	58	[182]
Thermal inactivation of peroxidase and lipoxygenase	Green asparagus	Biphasic 1st	70–95	43–53	[182]
Thermal inactivation of peroxidase and lipoxygenase	Carrot	Biphasic 1st	70–95	83–86	[182]
Overall sensory characteristics	Vegetable puree	1st	110–134	125–167	[183]
Ascorbic acid degradation	Mushroom	1st order with two degradation mechanisms	110–140	46.36–49.57	[184]
Green color degradation	Coriander leaves	1st	50–110	21.1–29.3	[82, 83]
Color parameter A	Spinach	Zero-order	65–85	117.7	[57]
B	Spinach	Zero-order	65–85	13.2	[57]
Δ*E*	Spinach	Zero-order	65–85	199.1	[57]
Betanin	Beet	1st	61.5–100	76.2	[160]
Vulgaxanthin	Beet	1st	61.5–100	83.4	[160]
Chlorophyll degradation	Broccoli juice	1st	80–120	69	[58, 77]
Chlorophylls	Spinach	1st	94–132.2	79.5	[58, 77]
Chlorophylls a and b	Asparagus	1st	70–89	54.6	[91]
Chlorophylls a and b	Green beans	1st	80–148	72	[95]
Chlorophylls	Peas	1st	80–148	76	[95]
Chlorophylls	Peas	1st	90–122	102.4	[2]
Chlorophylls	Pea puree	1st	94–132.2	92	[2]
Chlorophyll a	Spinach	1st	127–148	66.9	[2]
Chlorophyll a	Spinach	1st	116–126	114.2	[59]
Chlorophyll b	Spinach	1st	127–148	34.8	[59]
Chlorophyll b	Spinach	1st	116–126	103.4	[59]
Texture analysis	Dry peas	1st	70–100	146.69	[185]
Texture degradation	Beans	1st	90–120	97.0	[4]
Texture softening	Asparagus (green)	1st	70–98	100.8	[91]
Texture softening	Asparagus (green)	1st	115	56.4	[32]
Texture softening	Beetroot	1st	104.4–121.1	65.3	[92]
Texture softening	Beans (black)	1st	98–127	148.6	[92]
Texture softening	Beans (brown)	1st	98–127	156.9	[92]
Texture softening	Brussels sprouts	1st	100–150	125.7	[2]
Texture softening	Carrot	1st	104.4–121.1	63.6	[92]
Texture softening	Dry white beans	1st	104.4–121.1	104.2	[92]
Texture softening	Peas	1st	90–122	94.6	[4]

**Table 3:** Kinetic factors for microbial inactivation.

Type of microorganism	Temperature (°C)	Medium/substrate	*D*-value (Min)	*Z*-value (°C)	Reference
Human norovirus surrogates					
* Murine norovirus (MNV) *	37, 50	RAW 264.7 (ATCC TIB-71)	769, 106	—	[17]
* Murine norovirus (MNV) *	37, 52	Raw pig slurry	2.6, 1.3	—	[186]
* Murine norovirus (MNV) *	56	RAW 264.7 (ATCC TIB-71)	3.5	—	[187]
* Murine norovirus (MNV) *	56	RAW 264.7 (ATCC TIB-71)	3.473	—	[188]
* Murine norovirus (MNV) *	57	Chicken thigh	4.48	4.58	[189]
* Murine norovirus (MNV) *	58	Chicken breast	2.56	4.69	[189]
* Murine norovirus (MNV) *	59	Whole chicken	1.27	4.64	[189]
* Murine norovirus (MNV) *	60	Chicken thigh	1.179	—	[189]
* Murine norovirus (MNV) *	60	RAW 264.7 (ATCC TIB-71)	13.7	—	[189]
* Murine norovirus (MNV) *	61	Chicken breast	0.57	—	[189]
* Murine norovirus (MNV) *	63, 72	RAW 264.7 (ATCC TIB-71)	0.43, 0.16	—	[187]
* Murine norovirus (MNV) *	63	Water	0.9	—	[190]
* Murine norovirus (MNV) *	63	Milk	0.7	—	[190]
* Murine norovirus (MNV) *	63	RAW 264.7 (ATCC TIB-71)	0.435	—	[188]
* Murine norovirus (MNV) *	72	Water	0.3	—	[190]
* Murine norovirus (MNV) *	72	Milk	0.5	—	[190]
* Murine norovirus (MNV) *	72	RAW 264.7 (ATCC TIB-71)	0.166	—	[188]
* Feline calicivirus (FCV) *	56	Crandell Rees feline kidney cells	6.71	—	[187]
* Feline calicivirus (FCV) *	50	Fetal rhesus monkey kidney	50.6	—	[17]
* Feline calicivirus (FCV) *	56	Crandell Rees feline kidney cells	6.715	—	[188]
* Listeria monocytogenes *	52	Pasteurized milk	31	—	[191]
* Listeria monocytogenes *	50	Minced beef	33.83	—	[192]
* Listeria monocytogenes *	52	Tryptic soy broth	20.1	7.05	[193]
* Listeria monocytogenes *	52	Fresh boiled milk	27	—	[191]
* Listeria monocytogenes *	52	Butter	44.64	6.71	[193]
* Listeria monocytogenes *	52	Double cream	71.72	5.83	[193]
* Listeria monocytogenes *	52	Half cream	105.1	5.32	[193]
* Listeria monocytogenes *	52	Butter	23.68	6.67	[193]
* Listeria monocytogenes *	52	Double cream	58.055	6.08	[193]
* Listeria monocytogenes *	52	Half cream	43.3	6.2	[193]
* Listeria monocytogenes *	55	Fresh boiled milk	3.3	—	[192]
* Listeria monocytogenes *	55	Ready-to-eat turkey bologna	6	—	[194]
* Listeria monocytogenes *	55	Pork patties	150.46 ± 19.06	—	[195]
* Listeria monocytogenes *	55	Chicken thigh	38.94	—	[196, 197]
* Listeria monocytogenes *	55	Leg meat	82.75	—	[196, 197]
* Listeria monocytogenes *	55	Turkey	33.11	—	[196, 197]
* Listeria monocytogenes *	55	Beef	36.91	—	[196, 197]
* Listeria monocytogenes *	55	Ground pork	15.72	5.77	[198]
* Listeria monocytogenes *	55	Ground pork modified	16.97	5.53	[198]
* Listeria monocytogenes *	55	Pork slurry	4.75	4.63	[198]
* Listeria monocytogenes *	56	Skim milk	9	5.2	[198]
* Listeria monocytogenes *	56	Sodium phosphate buffer	3	5.9	[198]
* Listeria monocytogenes *	57	Fresh boiled milk	15	—	[191]
* Listeria monocytogenes *	57	Pasteurized milk	23.5	—	[191]
* Listeria monocytogenes *	57.5	Pork patties	55.08 ± 3.85	—	[195]
* Listeria monocytogenes *	60	Salmon	3.55	—	[199]
* Listeria monocytogenes *	65	Tilapia meat	1.13	—	[200]
* Listeria monocytogenes *	60	Orange juice	0.43	—	[117]
* Listeria monocytogenes *	61.1	Egg yolk	2.3	6.35	[201]
* Escherichia coli *					
* Escherichia coli *	51.66	Raw cream	34.4	—	[202, 203]
* Escherichia coli *	51.66	Raw milk	28.2	—	[202, 203]
* Escherichia coli *	51.66	Ice cream mix	39.3	—	[202, 203]
* Escherichia coli *	51.66	Chocolate milk	32.2	—	[202, 203]
* Escherichia coli *	51.7	Chocolate milk	34.4	10.2	[202, 203]
* Escherichia coli *	51.7	Cream	32.2	10	[202, 203]
* Escherichia coli *	51.7	Ice cream milk	39.3	10.3	[202, 203]
* Escherichia coli *	51.7	Milk	28.2	10.2	[202, 203]
* Escherichia coli *	51.7	Ground beef	115.5 (30.5% fat)	—	[194]
* Escherichia coli *	51.7	Skim milk concentrated	49.3	4.9	[204]
* Escherichia coli *	52.4	Skim milk concentrated	33.3	4.6	[204]
* Escherichia coli *	53	Skim milk concentrated	29.2	6.3	[204]
* Escherichia coli *	54	Egg white	1.82	3.98	[205]
* Escherichia coli *	54	Whole egg	9.1	3.95	[205]
* Escherichia coli *	54.44	Raw cream	10	—	[202, 203]
* Escherichia coli *	54.44	Raw milk	5.1	—	[202, 203]
* Escherichia coli *	54.44	Ice cream mix	15.2	—	[202, 203]
* Escherichia coli *	54.44	Chocolate milk	10.4	—	[202, 203]
* Escherichia coli *	55	Skim milk concentrated	23.5	7.9	[204]
* Escherichia coli *	55	Pork patties	32.11 ± 6.58	—	[195]
* Escherichia coli *	55	Apple cider	9.66	—	[206]
* Escherichia coli *	55	Minced beef	21.13	5.98	[207]
* Escherichia coli *	58	Apple cider	1.44	—	[206]
* Escherichia coli *	58	Brain heart infusion broth	5.6	4.7	[208]
* Salmonella *					
* Salmonella *	49.2	Alcohol-free beer	11.5	4.36	[209]
* Salmonella *	49.2	Lager	0.504	6.54	[209]
* Salmonella *	51.4	Skim milk concentrated	49	4	[204]
* Salmonella *	51.7	Skim milk concentrated	59.8	4.6	[204]
* Salmonella *	52.8	Skim milk concentrated	48.5	6	[204]
* Salmonella *	53	Skim milk concentrated	20.4	4.1	[204]
* Salmonella *	53.3	Skim milk concentrated	41.7	6.2	[204]
* Salmonella *	54	Egg white	1.51	4.03	[205]
* Salmonella *	54	Whole egg	5.7	4.08	[205]
* Salmonella *	55	Egg white product	0.73	4.7	[210]
* Salmonella *	55	Egg yolk	9.06	3.6	[210]
* Salmonella *	55	Whole egg modified	4.21	6.1	[210]
* Salmonella *	55	Whole egg product	6.05	3.8	[210]
* Salmonella *	55	Egg white product	1.08	4.4	[210]
* Salmonella *	60	Skim milk	3.6	8	[211]

**Table 4:** Kinetic factors affecting overall sensory quality of thermally processed vegetables.

Type of product	Temperature range *T* (°C)	Activation energy (KJ/mol)	Reaction rate, *k*_*T*_ (×10^3^/s)	Thermal destruction Rate, *D*_*T*_ (10^−3^ s)	*Z*-value (°C)	Reference
Peas	100–121	81.6	16	0.15	32.2	[94]
Green beans	84–116	171.6	38	0.06	15.6	[94]
Green beans	80–148	104	10	0.20	28.8	[94]
Corn whole kernel	80–148	94.6	70	0.26	31.7	[94]
Corn whole kernel	100–121	67	16	0.15	36.6	[212]
Carrots	80–116	160	27	0.084	16.7	[212]
Broccoli	100–121	54.4	8.7	0.26	44.4	[212]
Beetroot	80–110	142	19	0.12	19	[212]
Vegetable puree	110–134	125–167	—	—	18–24	[183]
Tomato sauce	110–134	111–187	—	—	16–27	[183]

**Table 5:** Kinetic parameters associated with chlorophyll and color degradation in mint and coriander at different pH and temperatures.

Product	Temperature (°C)	Form/size	Parameters	*k* (min^−1^) × 10^−3^	*E*_*a*_ (kJ/mol)	Reference
Mint						
pH						
4.5	80–100	Puree	Chlorophyll degradation	0.0111–0.0228	41.606	[213]
5.5	105–145	Puree	Chlorophyll degradation	0.0228–0.0604	33.785	[213]
6.5	105–145	Puree	Chlorophyll degradation	0.0384–0.0791	20.208	[213]
7.5	105–145	Puree	Chlorophyll degradation	0.0270–0.0465	16.569	[213]
8.5	105–145	Puree	Chlorophyll degradation	0.0274–0.0654	28.746	[213]
Coriander						
4.5	80–100	Puree	Chlorophyll degradation	0.0045–0.0233	83.24	[88]
5.5	105–145	Puree	Chlorophyll degradation	0.00633–0.0858	95.29	[88]
6.5	105–145	Puree	Chlorophyll degradation	0.0217–0.0698	38.48	[88]
7.5	105–145	Puree	Chlorophyll degradation	0.0261–0.0365	11.81	[88]
8.5	105–145	Puree	Chlorophyll degradation	0.0168–0.04918	40.47	[88]

**Table 6:** Kinetic parameters associated with texture degradation in asparagus, peas, beans, and dry beans.

Product	Temperature (°C)	Form/size	Parameters	*k* (min^−1^) × 10^−3^	*E*_*a*_ (kJ/mol)	Reference
Asparagus						
Asparagus	70–98	Spears	Texture degradation	0.0034–0.0618	24.5	[91]
Mushrooms	70–100	Strip	Hardness	0.152–0.269	15.22	[214]
Mushrooms	70–100	Strip	Adhesiveness	0.068–0.269	38.77	[214]
Mushrooms	70–100	Strip	Chewiness	0.129–0.178	0.70	[214]
Mushrooms	70–100	Strip	Cohesiveness	0.105–0.157	11.96	[214]
Mushrooms	70–100	Strip	Gumminess	0.028–0.237	59.64	[214]
Peas	120	Whole peas	Hardness	7.85	89.9	[4]
Beans	90	Whole beans	Hardness	7.64	97.0	[4]
Dry peas	70–100	Whole peas	Peak compression force (cook chill)	0.0017–0.1114	146.69	[185]
Dry peas	70–100	Whole peas	Peak compression force (*sous vide*)	0.0015–0.0534	125.78	[185]

**Table 7:** Kinetic parameters associated with color degradation in vegetables.

Product	Temperature (°C)	Reaction rate *k* (min^−1^) × 10^−3^	*E*_*a*_ (kJ/mol)	Thermal destruction rate *D* (×10^−3^ s)	*Z*-value	Reference
Asparagus	70	0.0029 ± 0.0002	12.9 ± 0.4	—	—	[91]
Asparagus	80	0.0050 ± 0.0002		—	—	[91]
Asparagus	90	0.0087 ± 0.0005		—	—	[91]
Asparagus	98	0.0130 ± 0.0010		—	—	[91]
Asparagus	70	0.0032 ± 0.0002		—	—	[91]
Asparagus	80	0.0054 ± 0.0004		—	—	[91]
Asparagus	90	0.0069 ± 0.0006		—	—	[91]
Asparagus	98	0.0167 ± 0.0010		—	—	[91]
Asparagus	70–89	0.11	54.6	—	—	[91]
Peas	90	8.22	85.4	—	—	[4]
Peas	80–148	1.5	76	1.50	39.4	[95]
Peas	121–148	1.23	67.9	31.1	42.9	[89]
Peas	90–122	0.09	102.4	25.8	26.4	[4]
Pea puree	94–132.2	0.34	92	6.90	32.5	[4]
Spinach	127–148	0.10	66.9	0.21	51.1	[4]
Spinach	116–126	2.8	114.2	0.82	26.2	[59]
Spinach	127–148	5	34.8	0.46	98.3	[59]
Spinach	116–148	1.36	103.4	1.70	29	[59]
Spinach	94–132.2	0.23	79.5	9.80	17.7	
Carrot betanin	61.5–100	1.8	76.2	1.28	36.5	[215]
Carrot vulgaxanthin	61.5–100	1.96	83.4	1.17	33.4	[215]
Carrot vulgaxanthin	61.5–100	2.2	64.5	1.05	43.1	[215]
Tomato puree	50–120	0.0004–0.0028	—	—	—	[79]
Mustard leaves	75–115	—	36.42	—	—	[82, 83]

**Table 8:** Kinetic parameters for textural softening of vegetables.

Product	Reaction constant *k* (min^−1^) × 10^−3^	Activation energy (kcal/mol)	Reference
Asparagus	0.005–0.0983	24.5	[91]
Mushroom	0.028–1.161	0.70–59.64	[214]
Peas	7.64	89.9	[4]
Beans	7.64	97.0	[4]
Dry peas	0.0004–0.1319	112.43–146.69	[185]
Asparagus (green)	3.65	56.4	[32]
Beetroot	7.13	65.3	[92]
Carrot	3.9	63.6	[92]
Dry white beans	66	104.2	[92]
Whole kernel corn	0.384	19.5	[94]
Peas	1.0	22.5	[94]
Cut green beans	0.576	22.0	[94]
Peas (extrusion)	0.250	18.5	[96]
Carrot (Rothild, 3 mm)	0.494	27.2	[101]
Carrot (Kundulus, 3 mm)	0.259	22.0	[101]
Carrot (Rubika, 3 mm)	0.422	28.0	[101]
Potatoes	0.516	28.0	[102]
Canned black beans (untreated)	0.057	19.1	[39]
Canned black beans	0.978	31.3	[39]
Canned black beans	0.983	38.9	[39]

**Table 9:** Effect of temperature and solids content on degradation of ascorbic acid in grape juice and concentrate. Adapted from [215].

Solids content (°Bx)	Temperature (°C)	Rate constant *K* × 10^3^ (min^−1^)	*E*_*a*_ (kcal/mol)	Half life *T*_1/2_ (min)	Correlation coefficient-*r*	Arrhenius equation coefficient ln(*K*_0_)
11.2	61	1.276	4.98	543.2	0.968	0.845
	80	1.899		365.0	0.983	
	95	2.503		276.9	0.983	
	96	2.642		264.2	0.968	
31.2	60	1.349	5.24	514.0	0.962	1.297
	75	1.874		369.7	0.987	
	82	2.165		320.1	0.978	
	91	2.701		265.8	0.89	
47.1	61	1.430	6.69	485.0	0.981	3.531
	80	2.460		281.8	0.988	
	90	3.121		221.9	0.982	
	96	3.777		183.8	0.967	
55.0	61	1.618	8.60	428.4	0.975	6.528
	75	2.715		255.3	0.985	
	81	3.348		207.0	0.986	
	91	4.712		147.1	0.994	
62.5	68	3.022	11.28	229.4	0.973	10.802
	76	4.261		162.7	0.964	
	81	5.365		129.2	0.972	
	96	10.680		64.9	0.961	

**Table 10:** Effect of temperature and solids content on anaerobic degradation of ascorbic acid in orange serum and in whole orange juice. Adapted from [216].

Solids content (°Bx)	Temperature (*K*)	Orange serum rate constant *K* × 10^4^ (min^−1^)	Orange serum *E*_*a*_ (kcal/mol, temp. 70.3–97.6°C)	Orange juice rate constant *K* × 10^4^ (min^−1^)	Orange juice *E*_*a*_ (kcal/mol, temp. 70.3–97.6°C)	Orange serum Reaction constant ln (*A*_0_, temp. 70.3–97.6°C)	Orange juice reaction constant ln (*A*_0_, temp. 70.3–97.6°C)
12.7	343.5	0.43	29.2	0.45	30.7	32.8	35.2
37.3	343.5	0.91	28.6	1.45	23.3	32.5	25.3
55.8	343.5	1.16	30.3	1.29	28.7	35.5	33.2
80.6	343.5	2.89	30.8	2.74	27.5	37.2	32.2
12.7	355.2	2.34	29.2	2.99	30.7	32.8	35.2
37.3	355.2	3.31	28.6	4.16	23.3	32.5	25.3
55.8	355.2	6.23	30.3	6.17	28.7	35.5	33.2
80.6	355.2	18.4	30.8	11.2	27.5	37.2	32.2
12.7	364.4	5.47	29.2	8.86	30.7	32.8	35.2
37.3	364.4	9.27	28.6	10.6	23.3	32.5	25.3
55.8	364.4	17.7	30.3	18.2	28.7	35.5	33.2
80.6	364.4	46.9	30.8	34.5	27.5	37.2	32.2
12.7	370.8	10.3	29.2	11.3	30.7	32.8	35.2
37.3	370.8	20.3	28.6	17.4	23.3	32.5	25.3
55.8	370.8	29.6	30.3	26.6	28.7	35.5	33.2
80.6	370.8	80.0	30.8	48.9	27.5	37.2	32.2

**Table 11:** Lycopene contents of different fruits and vegetables.

Product	Lycopene content (mg/100 g wet basis)	Reference
Carrot	0.65–0.78	[217, 218]
Sweet potato	0.02–0.11	[217, 218]
Pumpkin	0.38–0.46	[217, 218]
Rosehip puree	0.68–0.71	[217, 218]
Fresh tomato fruit	0.72–20	[217, 218]
Watermelon	2.3–7.2	[217, 218]
Guava (pink)	5.23–5.50	[217, 218]
Grapefruit (pink)	0.35–3.36	[217, 218]
Papaya	0.11–5.3	[217, 218]
Apple pulp	0.11–0.18	[217, 218]
Apricot	0.01–0.05	[217, 218]
